# On reduced-order modeling of drug dispersion in the spinal canal

**DOI:** 10.1186/s12987-025-00657-6

**Published:** 2025-07-02

**Authors:** F. J. Parras-Martos, A. L. Sánchez, C. Martínez-Bazán, W. Coenen, C. Gutiérrez-Montes

**Affiliations:** 1 Área de Mecánica de Fluidos, Departamento de Ingeniería Mecánica y Minera, Universidad de Jaén, Jaén, 23071 Spain; 2 Andalusian Institute for Earth System Research, Universidad de Jaén, Campus Las Lagunillas s/n, Jaén, 23071 Spain; 3 Department of Mechanical and Aerospace Engineering, University of California San Diego, La Jolla, 92093 USA; 4 Área de Mecánica de Fluidos, Departamento de Mecánica de Estructuras e Ingeniería Hidráulica, Universidad de Granada, Granada, 18071 Spain; 5 Andalusian Institute for Earth System Research, Universidad de Granada, Avenida del Mediterráneo s/n, Granada, 18006 Spain; 6 Grupo de Mecánica de Fluidos, Departamento de Ingeniería Térmica y de Fluidos, Universidad Carlos III de Madrid, Leganés, 28911 Spain

**Keywords:** Biomedical fluid dynamics, Cerebrospinal fluid flow, Intrathecal drug delivery, Lagrangian motion

## Abstract

The optimization of intrathecal drug delivery procedures requires a deeper understanding of flow and transport in the spinal canal. Numerical modeling of drug dispersion is challenging due to the disparity in time scales: dispersion occurs over 1 hour, while cerebrospinal fluid pulsations driven by cardiac motion occur on a 1-second scale. Patient-specific predictions in clinical settings demand simplified descriptions that focus on drug-dispersion times, bypassing the rapid concentration oscillations caused by cyclic motion. A previously derived reduced-order model involving convective transport driven by mean Lagrangian drift is tested here through comparisons with MRI-informed direct numerical simulations (DNS) of drug dispersion in a cervical-canal model featuring nerve rootlets and denticulate ligaments. The comparisons demonstrate that the reduced model is able to describe precisely drug transport, enabling drug-dispersion predictions at a fraction of the computational cost involved in the DNS. Approximate descriptions assuming convective transport to be governed by the mean Eulerian velocity are found to significantly underpredict drug dispersion, highlighting the critical role of mean Lagrangian motion. Our results also confirm the substantial influence of microanatomical features on drug dispersion, consistent with earlier analyses. A key additional finding from the DNS is that molecular diffusion has a negligible impact on drug dispersion, with the mean drift of fluid particles primarily dictating the evolution of the drug distribution—an insight valuable for future modeling efforts.

## Background

**Fig. 1:**
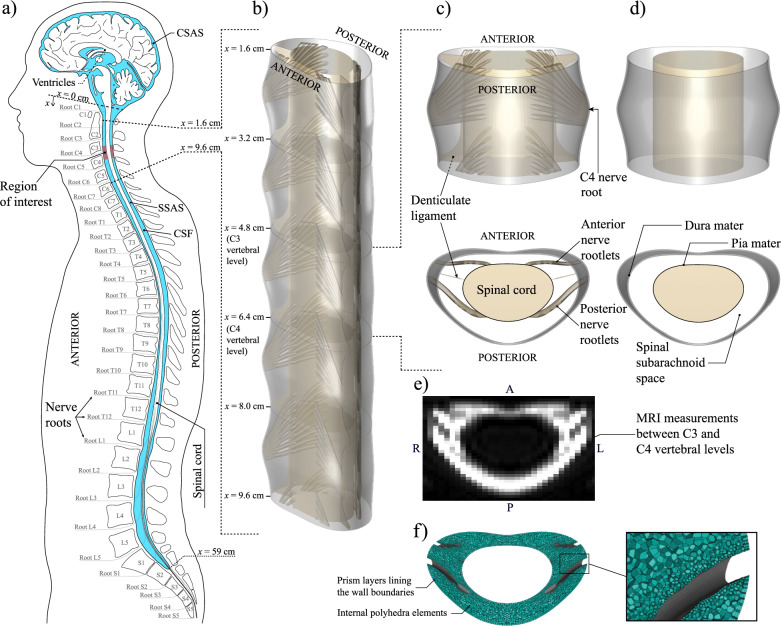
Illustration of the spinal canal and the microanatomical features herein considered, i.e. nerve rootlets and denticulate ligaments. **a**) Sketch of the SAS showing in red the region of interest in the present study. **b**) Model of the anatomy of the spinal canal constructed by stacking five identical vertebral sections, including nerve rootlets and denticulate ligaments, between axial positions $x=1.6 \, \text{cm}$ and $x=9.6 \, \text{cm}$ measured from the foramen magnum. **c**) Anatomically realistic (obstructed) geometrical model between the C3 and C4 vertebral levels, shown in posterior and top views. **d**) Patent (unobstructed) geometrical model between the C3 and C4 vertebral levels, shown in posterior and top views. **e**) Cross-sectional MRI view of the spinal canal at the midpoint between the C3 and C4 vertebral levels. **f**) Cross section of the computational mesh of the cervical segment shown in **c**), and detail of the mesh in the vicinity of a nerve rootlet and the dura membrane

The cerebrospinal fluid (CSF) is a colorless Newtonian liquid that fills the ventricles of the brain and the subarachnoid space (SAS) surrounding the brain and the spinal cord, as shown in Fig. 1a. It is mainly produced in the choroid plexi within the cerebral ventricles [1, 2], and it is absorbed into the bloodstream by the arachnoid villi [2]. In addition to the secretion-absorption pathway, the CSF describes a complicated motion characterized by a strong pulsating component, synchronized with the cardiac [3, 4] and respiratory [5–7] cycles. Superimposed on this oscillatory flow is a smaller steady component [8–10], resulting from the nonzero time-averaged flux of the pulsatile motion.

CSF motion plays an important physiological role in the transport of nutrients and metabolic waste in the central nervous system, primarily during sleep. Its deregulation has been related to the development of neurodegenerative disorders such as Alzheimer’s disease, Parkinson’s disease, and cerebral amyloid angiopathy [11, 12]. Furthermore, CSF flow is instrumental in the dispersion of drugs released directly in the spinal subarachnoid space (SSAS) [10], a procedure called intrathecal drug delivery (ITDD) that is widely used to administer therapeutic drugs [13–15] to treat severe pain and spasticity [16–19] as well as neuraxial pathologies (infection [20]/cancer [21]) or neurodegenerative processes (e.g., Somatomotor Atrophy [22]/Amyotrophic Lateral Sclerosis [19]). The focus of the present analysis is on the description of flow and transport in the SSAS, the ultimate objective being the development of reduced-order models that can provide accurate predictions of drug dispersion at a minimal computational cost for application in clinical settings.

Patterns of CSF flow and transport in the SSAS are strongly dependent on the specific physiology and anatomy of the subject [23], including the morphology of the pia and dura surfaces, the eccentricity of the spinal cord, and the so-called microanatomy [24], which encompasses structures such as nerve roots, denticulate ligaments, and trabeculae [25]. To improve the understanding of these factors, several studies have been conducted in the literature. Loth et al. [26] studied numerically the oscillatory motion of CSF in an anatomically relevant spinal canal consisting of idealized pia mater and dura mater membranes, modeled on the basis of magnetic resonance imaging (MRI). However, their model did not account for finer anatomical structures such as nerve roots, denticulate ligaments, and trabeculae. They concluded that convective effects dominate the flow and that the velocity field is sensitive to spinal cord eccentricity, their results being only qualitatively compared with phase-contrast MRI velocity measurements. Based on this idea, Stockman [27, 28] modeled oscillatory CSF flow and solute transport in a canonical geometry of the spinal canal, modeled as a circular annulus, incorporating idealized trabeculae, nerve roots, and denticulate ligaments. These studies, using a lattice Boltzmann method, showed that microanatomical features increase significantly the longitudinal dispersion, and also demonstrated that the associated enhanced dispersion is dominated by advective effects, with diffusion being largely inconsequential. Hsu et al. [29] simplified the three-dimensional SSAS model by assuming rotational symmetry, reducing it to a two-dimensional model, which did not include the presence of microanatomical elements. This allowed them to numerically quantify the effects of frequency and oscillation amplitude on drug dispersion, using results from MRI and CINE-phase-contrast MRI measurements as input. Linninger and coworkers [30–33] developed a closed geometric model of the SAS based on MRI reconstructions of the craniospinal space, but neglecting trabeculae. They implemented moving walls to couple cardiovascular dynamics with CSF oscillations, and validated the model predictions of CSF velocity magnitude and stroke volume through comparisons with CINE-phase-contrast MRI. Following this previous work, Tangen et al. [34] numerically investigated the effect of idealized trabeculae and nerve roots on flow and drug dispersion, modeling the anterior and posterior nerve roots as a single, large-diameter cylindrical structure reconstructed from medical images. After simulating the oscillatory flow and validating the results with MRI measurements, they found that trabeculae increase flow resistance by a factor of two to three, while nerve roots create recirculation patterns and significantly accelerate drug transport. Pahlavian et al. [35] investigated the effect of idealized nerve roots and denticulate ligaments in the cervical region of the spinal canal, the latter reconstructed from MRI images. The oscillatory flow of CSF was numerically solved, providing insights on the role of the microanatomy in the driving pressure gradients and in the formation of recirculation patterns.

The above numerical models implement transient oscillatory simulations to solve the flow field or drug transport. Since the streamwise dispersion of the drug requires a large number of oscillatory cycles, the computational cost of this approach makes its implementation prohibitively expensive. Kuttler et al. [36] addressed this issue by proposing a simplified transport equation. Their analysis, not accounting for the microanatomy, began by solving the CSF oscillatory flow using a finite-volume method. A cross-sectional averaged mass flow was used in the cervical region as a periodic inlet condition to represent cranial blood volume changes, while effects of breathing were incorporated through contractions and expansions of the SSAS. The time-averaged Eulerian velocity field was calculated over one respiratory cycle, providing a stationary velocity field with a non-zero mean flow in the canal. They used this new velocity field in computations of drug transport, a computational approach that significantly reduces computational time.

The effect of nerve roots on the time-averaged Eulerian velocity was investigated by Khani et al. [24], who solved the flow by modeling the spinal canal with a deformable dura mater. They found that the presence of nerve roots increases significantly the magnitude of the time-averaged Eulerian velocity. In a later study, Khani et al. [37] followed the approach of Kuttler et al. [36] by replacing the instantaneous velocity with the time-averaged Eulerian velocity when writing the convective term in the solute transport equation. Their computations, neglecting effects of molecular diffusion, revealed that the injection location and protocol significantly affect the spatial distribution of the drug over time. More recently, Wang et al. [38] investigated numerically differences between time-averaged Eulerian velocity (calculated by cycle averaging the oscillatory velocity field) and mean Lagrangian velocity (evaluated numerically using Lagrangian particle tracking) in a model of the spinal subarachnoid space. Their results demonstrated that microanatomical features amplify the disparities between these two velocity fields.

Of particular relevance to the present study is a series of theoretical papers addressing flow and transport in the spinal canal [10, 39, 40]. This previous work models the SSAS as a compliant annular space of slowly varying cross section closed at one end and subject to an oscillating pressure at the other end. The analysis exploits simplifications arising from the disparity of scales present in the problem. Specifically, the asymptotic description employs expansions in terms of a small parameter, $\varepsilon$, representing the ratio of the characteristic stroke length to the characteristic streamwise length associated with the canal morphology. The main result of the asymptotic description is a reduced transport equation for the solute concentration, *c*, given in dimensionless form in equation 3.12 in Lawrence et al. [10]. When written in dimensional form, the equation reads

1 $$\begin{aligned} \frac{\partial c}{\partial t} + {\boldsymbol{v}}_L \cdot \nabla c = \kappa \nabla ^2 c \end{aligned}$$

where *t* represents the time, $\kappa$ is the drug diffusivity and ${\boldsymbol{v}}_L({\boldsymbol{x}})$ denotes the mean Lagrangian velocity vector of the fluid particles, a time-independent function of the position ${\boldsymbol{x}}$. As explained by Lawrence et al. [10], in the limit $\varepsilon \ll 1$ considered in the analysis, this mean Lagrangian velocity can be determined by linearizing the velocity when computing the small cyclic displacement experienced by a fluid particle as it moves in the close vicinity of a given point. The result can be expressed as the sum of two separate contributions

2 $$\begin{aligned} {\boldsymbol{v}}_L=\langle {\boldsymbol{v}} \rangle +\left\langle \int ({\boldsymbol{v}}-\langle {\boldsymbol{v}} \rangle ) \text{d}t \cdot \nabla ({\boldsymbol{v}}-\langle {\boldsymbol{v}} \rangle ) \right\rangle , \end{aligned}$$

where ${\boldsymbol{v}}({\boldsymbol{x}},t)$ is the instantaneous value of the CSF velocity vector and

3 $$\begin{aligned} \langle \star \rangle =\frac{1}{T} \int _t^{t+T} \star \, \text{d}t \end{aligned}$$

is the cycle-averaging operator, with *T* representing the flow-oscillation period. The first term on the right-hand side of (2) is the cycle-averaged Eulerian velocity (i.e. the so-called streaming velocity), $\langle {\boldsymbol{v}} \rangle$, while the second term, ignored in the previous modeling work of Kuttler et al. [36] and Khani et al. [37], is the Stokes drift, a kinematic effect resulting from the spatial non-uniformity of the flow that can be computed in terms of the purely oscillatory component of the velocity ${\boldsymbol{v}}-\langle {\boldsymbol{v}} \rangle$. It is important to note that the expression given in (2) is strictly valid only for $\varepsilon \ll 1$. For order-unity values of $\varepsilon$ the mean Lagrangian velocity must be evaluated numerically by tracking the fluid particles over an oscillatory cycle, that being the approach adopted by Wang et al. [38].

The description (1) was combined by Coenen et al. [23] with anatomical T2-weighted images and phase-contrast MRI measurements of CSF velocity, showing recirculating patterns of CSF bulk flow in all subjects studied. More recently, Alaminos-Quesada et al. [41, 42] extended the reduced model to incorporate effects of buoyancy, arising in ITDD procedures when the drug density differs from the CSF density [22].

It is worth noting that in the reduced transport equation (1), derived from a rigorous asymptotic analysis [10], convective transport is driven by the steady mean Lagrangian drift. This feature of the model enables the description to focus directly on the long-time drug dispersion, circumventing the computation of the concentration fluctuations arising from the velocity oscillations. As a result, associated computational times are reduced by several orders of magnitude relative to those involved in Direct Numerical Simulations (DNS), potentially facilitating use of the model in future subject-specific studies in clinical settings.

The accuracy of the transport equation (1) has been tested through DNS [40] and experiments [43], but only under idealized conditions—specifically, doubly-slender, unobstructed geometries (neglecting microanatomy) and harmonic intracranial pressure waveforms with small stroke volumes ($\varepsilon \ll 1$). For the first time, the present study extends and evaluates this reduced-order model for drug dispersion under realistic CSF flow conditions and anatomically accurate SSAS geometries. Our analysis focuses on the cervical spinal canal and employs an MRI-based anatomical model spanning five vertebral segments. This model includes key microanatomical features such as nerve rootlets—fine nerve filaments that emerge from the spinal cord and merge to form nerve roots—and denticulate ligaments (see Fig. 1b). Within this framework, we compute the long-term dispersion of drugs driven by the mean Lagrangian motion that persists beyond the purely oscillatory cardiac-driven motion that dominates the flow in the cervical SSAS.

Since the rigorous derivation of (1) relies on the stroke length being much smaller than the characteristic longitudinal length of the problem, there is interest in testing the accuracy of the model in the presence of microanatomical features, such as nerve rootlets and denticulate ligaments, whose characteristic size and separation distance are typically comparable to the stroke length. The reduced-order model is implemented following a three-step method in which the Eulerian velocity is first obtained from MRI-informed DNS of the flow field over one cycle, and is subsequently employed to evaluate the mean Lagrangian motion, which in turn is used in the integration of the reduced-order transport equation (1) in the long time scale for a particular initial drug distribution. The results are compared with those of high-fidelity DNS of the complete unsteady transport equation, which fully resolve the fast fluctuations of drug concentration associated with the oscillatory CSF motion during each cardiac cycle, over many of such cycles. As shown below, the comparison reveals that the reduced-order model is able to describe with outstanding accuracy the spatiotemporal evolution of the solute concentration at a fraction of the computational time required by the DNS. Previously proposed reduced transport descriptions assuming that convective transport is driven by the mean Eulerian velocity will be shown to significantly underpredict drug dispersion, thereby underscoring the key role of the mean Lagrangian motion. The computations below will also confirm the important role of microanatomical features on drug dispersion, in agreement with previous analyses [27, 28, 34, 37]. An important additional finding of the DNS, relevant in future modeling efforts, is that molecular diffusion has a negligible effect on drug dispersion, so that it is the mean drift of the fluid particles that effectively determines the evolution of the drug distribution.

## Methods

In this section, we detail the methodology employed to evaluate the predictive capability of the reduced-order model (1) for drug dispersion within the spinal canal. The focus is placed on drug dispersion in the cervical region, where cardiac-driven oscillatory motion dominates, and where the associated CSF stroke length is comparable to the intervertebral distance and to the separation distance between nerve roots and denticulate ligaments, such that the reduced-order model is placed outside its strict range of operation. Sections Magnetic resonance imaging and A model of the cervical canal explain how MR imaging and detailed anthropometric measurements were employed to construct a realistic anatomical model by stacking five cervical vertebrae. The resulting geometry was meshed, and high-fidelity DNS were carried out to generate the benchmark results used to validate the reduced-order model. It was found computationally advantageous to separate the numerical simulations in two steps. First, the time-periodic Eulerian oscillatory velocity field was calculated, using input from in-vivo phase-contrast MRI velocity measurements, as explained in sections Acquisition of the oscillatory flow rate and Fluid flow. Then, the drug transport problem was solved, imposing the previously obtained time-periodic Eulerian velocity field (section Solute transport), starting from a canonical, bolus-like, initial drug distribution. The same drug-dispersion problem was then tackled with the reduced-order model, as detailed in section Implementation of the reduced model. Its implementation was semi-numerical in the sense that the previously computed time-periodic Eulerian velocity field was re-used to evaluate the mean Lagrangian motion that appears in the advective term of the reduced-order transport equation (1).

### Magnetic resonance imaging

The anatomy and CSF flow rate of the cervical canal of a 36-year-old man in presumed good health were acquired using MRI techniques, similar to those employed in our previous investigation [23]. The study was approved by the institutional review board, and written informed consent was obtained from the subject before MR imaging. A neuroradiologist reviewed the MR images to exclude spinal pathologies. The MR measurements provided the three-dimensional structure of the pia mater and dura mater and the location of the spinal nerves, and allowed for the measurement of CSF flow velocities across the cardiac cycle.

Imaging employed a 3T Magnetom Prisma Fit MRI scanner (Siemens) with 64-channel head and neck coils alongside a 32-channel spine coil. High-resolution images of the spinal canal were obtained using a 3D T2-weighted sagittal SPACE sequence, configured with TR = $\mathrm {1500 \, ms}$, TE = $\mathrm {231 \, ms}$, bandwidth = $\mathrm {504 \, Hz/pixel}$, 1.4 averages, and in-plane resolution of $0.8 \times 0.8$ interpolated to $0.4 \times 0.4$ $\mathrm {mm^2}$, with 64 slices per block and a slice thickness of $\mathrm {0.8 \, mm}$. At selected sites within the spinal canal, CSF flow-velocity data were acquired using a cardiac-gated, 2D phase-contrast MRI sequence perpendicular to the spinal canal longitudinal axis. Key imaging parameters included a 15-degree flip angle, FOV of $160 \times 160$ $\mathrm {mm^2}$, matrix size of $256 \times 205$, an in-plane resolution of $0.625 \times 0.78$ reconstructed to $0.625 \times 0.625$ $\mathrm {mm^2}$, and slice thickness of $\mathrm {10 \, mm}$.

### A model of the cervical canal

The computational strategies employed in the present work require different models of the cervical spinal canal. Drug dispersion is to be computed within a five-segment section of the SSAS, constructed by repeating a single vertebral segment five times, as illustrated in Fig. 1b. The morphology of the individual segment was defined using the SSAS region between cervical levels C3 and C4. This spatially periodic setup significantly reduces computational cost by enabling the simulation of CSF oscillatory flow within a single representative segment. Because the size of many microanatomical aspects of the spinal canal is below medical image resolution, the generation of the model followed a two-step process. In the first step, we used MR images to determine the shape of the dura mater and pia mater that define the lateral boundaries of the cervical SSAS. In the second step, the annular space was populated by obstacles representing nerve rootlets and denticulate ligaments (the microanatomical features considered in this work) using available anthropometric data gathered in previous post-mortem studies [44–46]. Since the density of trabecular structures is very low in the cervical region, as demonstrated in post-mortem imaging studies (see, e.g., Fig. 34.2 of Reina et al. [25]), their presence was neglected in generating the model.

The image collections were segmented in axial orientation using the threshold-based semi-automatic segmentation tool of the open-source program ITK-SNAP (Version 4.2.0; www.itksnap.org; [47]). MATLAB (R2024a; MathWorks, Massachusetts) was used to extract the 3D positions of the pia mater and dura mater. SolidWorks (Version 2022; Dassault Systèmes SolidWorks Corporation, Massachusetts) was employed to construct a control volume in STEP format between the cervical levels C3 and C4, based on the previously filtered coordinates of the pia mater and dura mater. Analysis shows that the vertebral levels C3 and C4 are located at a distance from the foramen magnum $x= 4.8$ cm and $x= 6.4$ cm, respectively (see Fig. 1a). The geometry near the C3 and C4 levels was slightly adjusted to ensure identical cross sections, as needed to enable section stacking. The volume of fluid contained in the vertebral section is $V_{\mathrm{C3-C4}}=\mathrm {1.875 \, cm^3}$, corresponding to a mean cross-sectional area of $\mathrm {1.172 \, cm^2}$. The shape of the resulting annular canal is shown in Fig. 1d. As explained below, this patent (unobstructed) geometry was used in numerical simulations to quantify the isolated effect of nerves and denticulate ligaments on flow and drug transport.

The modeling of the nerve rootlets was based on the study by Mendez et al. [44], who provided a precise description of the posterior and anterior spinal nerves in the SSAS of nine adult human cadavers. For the C4 nerve root, the authors find an average of eight nerve rootlets in the anterior part and nine in the posterior part (see Fig. 2I in Mendez et al. [44]). The average diameter of these nerve rootlets was calculated from the height of the anterior and posterior nerve roots when they exit the SSAS, given in Fig. 2P of Mendez et al. [44]. Although the authors refer to this height as the root diameter, in the cervical region the nerve rootlets that originate in the spinal cord are grouped approximately in pairs forming subbundles, and these are grouped vertically forming the nerve root. Consequently, the authors’ measurements in this area correspond to the sum of the diameters of the subbundles. By using this magnitude, it is possible to determine the diameter of the subbundles by dividing the height of the root by the number of subbundles, which for the anterior and posterior parts is approximately four, i.e. half the number of nerve rootlets (this can be seen in Fig. 2A of Mendez et al. [44]). Once the average diameter of the subbundles is known, it becomes possible to determine the average diameter of the nerve rootlets, given that the number of fibers forming the nerve rootlets remains constant. In other words, the total cross-sectional area of all the subbundles must be equal to the total cross-sectional area of all the nerve rootlets. In this way, by equating areas, it is possible to obtain an average value for the rootlet diameter, yielding $\mathrm {0.52 \, mm}$ and $\mathrm {0.8 \, mm}$ in the anterior and posterior sides, respectively. This value was verified by performing a scaling analysis of Fig. 2A of Mendez et al. [44], from which the height of the root is known and the relationship between it and the diameter of the different nerve rootlets can be calculated.

Additionally, the rostral to caudal distance reported in Fig. 2A of Mendez et al. [44] was used to determine the spacing between consecutive nerve rootlets on the surface of the pia mater, yielding an average value of $\mathrm {10.5 \, mm}$ for the anterior region and $\mathrm {12 \, mm}$ for the posterior region. We also used the transverse diameter of the spinal cord, the ventral thickness, and dorsal thickness shown in Fig. 2M of Mendez et al. [44], to establish relationships that allowed us to extrapolate these measurements to our geometric model, and thus determine the azimuthal location (relative to the center of the spinal cord) where the anterior and posterior nerve rootlets exit the cord. Finally, our MR images were used to determine the azimuthal location where the nerve roots intersect the surface of the dura membrane.

The modeling of the denticulate ligaments was based on detailed descriptions by Tubbs et al. [45] and Ceylan et al. [46]. These ligaments are characterized by their simple structure, consisting of a thin lamina in an axial arrangement that connects the pia mater to the dura mater along specific vertebral levels. Between these vertebral anchorage points, the ligament remains attached only to the pia mater, protruding slightly like a thin blade.

The nerve rootlets and denticulate ligaments were added to the unobstructed annular model shown in Fig. 1d, yielding the vertebral section shown in Fig. 1c, to be used in building an anatomically accurate model of the spinal canal. The resulting geometry compares favorably with that revealed in our MR study at the C3 and C4 vertebral levels, as can be seen by comparing the cross-sectional view of the spinal-canal model shown in the bottom panel of Fig. 1c with the MR image shown in Fig. 1e. We used ANSYS Fluent Meshing (2023 R1; Ansys Inc., Pennsylvania) to generate an unstructured polyhedral mesh for the computational domain shown in Fig. 1c, to be used in the numerical simulations of fluid flow and drug transport, Fig. 1f. We applied different face sizing to control the cell size and adapt it to the varying scales of the microanatomy. The selected polyhedral mesh had $2\times 10^5$ cells and $1.2 \times 10^6$ faces. The minimum orthogonality quality value was 0.383, which was enough considering the anatomical complexity. A mesh sensitivity study involving computations of oscillatory flow in a refined mesh containing $4\times 10^5$ cells found relative errors of velocity magnitudes smaller than $0.216\%$, thereby indicating that the selected mesh of $2\times 10^5$ cells was sufficiently accurate for computational purposes. As one of the objectives of this work is to compare the effect of nerve rootlets and denticulate ligaments on flow and transport in the spinal canal, a second hexahedral mesh of $4.5\times 10^4$ cells and $1.389\times 10^5$ faces was generated using the patent geometry shown in Fig. 1d. As explained in section Direct numerical simulations, the above two computational domains were used in computing the flow of CSF in a single segment. The drug transport problem was then solved in the extended domain, constructed by stacking the individual segment five times.

**Fig. 2:**
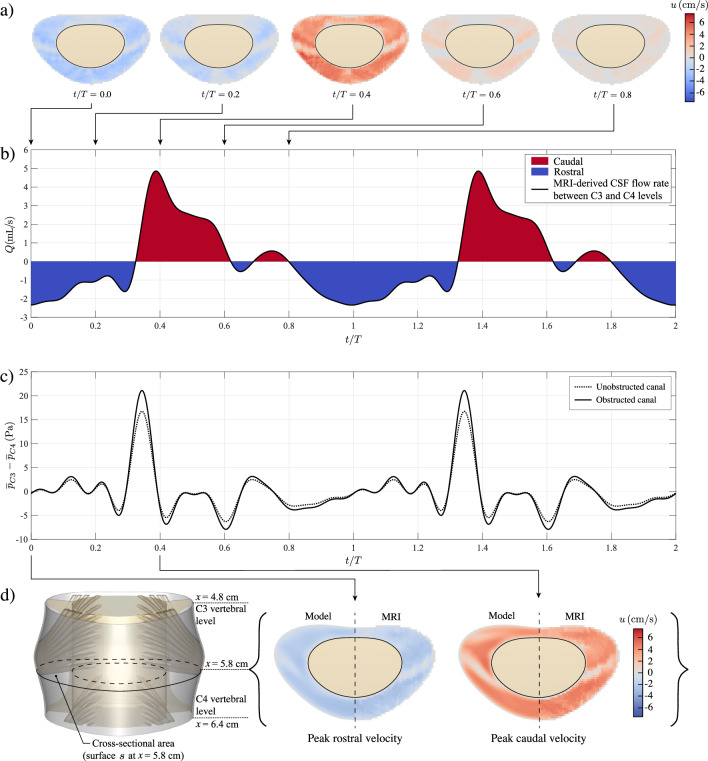
**a**) Contours of axial velocity, *u*, obtained from phase-contrast MRI measurements at different instants during the cardiac cycle in a cross-sectional area located at the midpoint between the C3 and C4 vertebral levels. **b**) Time evolution of the flow rate obtained from the measurements shown in **a**), where two full oscillatory cycles are displayed. **c**) Time evolution of the pressure difference between the C3 and C4 vertebral levels during two cardiac cycles considering both the patent geometry (dashed line) and the anatomically realistic geometry (solid line). **d**) Comparison of the axial velocity contours at $x= 5.8$ cm given by the oscillatory flow model (left half of the contour) and those obtained from the phase-contrast MRI measurements (right half of the contours) at the times of maximum magnitude of the rostral ($t=0$) and the caudal velocities ($t=0.4 \, T$), respectively. Symmetry respect to the mid-sagittal plane (dashed lines) has been considered in the model

### Acquisition of the oscillatory flow rate

The axial velocity between vertebral levels C3 and C4 was acquired using MRI velocimetry, as described above in section Magic resonance imaging. A total of 40 velocity samples were taken during each cardiac cycle. The velocity data were adjusted to the geometry through linear interpolation, aiming to improve accuracy near the pia mater and dura mater. The result of this processing can be seen in Fig. 2a, which shows the oscillatory axial velocity $u \, \mathrm {(cm/s)}$ across the section *s* indicated in Fig. 2d. Positive values (red) of the velocity correspond to caudal motion (downward), whereas negative values (blue) correspond to rostral motion (upward). The discrete velocity distribution, involving voxels of in-plane size 0.625 x 0.625 $\hbox {mm}^2$, exhibits a deficit in the regions occupied by the nerve rootlets in Fig. 2a and d. The resulting velocity was integrated across the canal section to provide the cyclic variation of the flow rate shown in Fig. 2b, where two full oscillatory cycles are considered. This waveform is to be employed to define the boundary conditions for the DNS of flow velocity, as described below in section Fluid flow, with the period *T* set equal to $T=1$ s in all computations.

### Direct numerical simulations

#### Fluid flow

The numerical simulations performed in this paper neglect density differences induced by the presence of the drug (see Alaminos-Quesada et al. [41, 42] for an account of buoyancy effects stemming from density variations). In this constant-density approximation, the velocity field can be determined independently of the solute concentration by integration of the continuity and momentum conservation equations

4 $$\begin{aligned} \nabla \cdot {\boldsymbol{v}} = 0, \quad \frac{\partial {\boldsymbol{v}}}{\partial t} + {\boldsymbol{v}} \cdot \nabla {\boldsymbol{v}} = -\frac{1}{\rho } \nabla p + \nu \nabla ^2{\boldsymbol{v}}, \end{aligned}$$

where ${\boldsymbol{v}}$ is the velocity vector, whose axial, transverse and azimuthal coordinates are *u*, *v* and *w*, respectively. In the momentum equation $\rho \simeq 1000 \, \mathrm{kg/m^3}$ and $\nu \simeq 0.7 \times 10^{-6} \, \mathrm{m^2/s}$ are the CSF density and kinematic viscosity [48–50] and *p* represents the pressure head (i.e., the sum of the static pressure and the hydrostatic pressure).

As explained in Appendix A, computations involving displacement of the dura mater to model the compliance of the cervical spinal canal revealed that its effect on the local distributions of velocity and associated time-averaged Eulerian velocity $\langle {\boldsymbol{v}} \rangle =(\langle u\rangle ,\langle v\rangle ,\langle w\rangle )$ is relatively small, so that in the first approximation a rigid model is sufficiently accurate for local evaluation of solute transport in the cervical region. Correspondingly, the boundary conditions for integration of (4) include a no-slip condition (${\boldsymbol{v}} = 0$) on the bounding solid surfaces (pia mater, dura mater, nerve rootlets, and denticulate ligaments). Since the spinal canal model was built by stacking identical vertebral sections, the resulting velocity field is spatially periodic and its determination only requires consideration of a single vertebral section, depicted in Fig. 1c and d, with a periodic boundary condition imposed on the velocity at both the inlet (C3) and the outlet (C4) boundaries. This approximation leads to identical velocity profiles at the two open boundaries, thereby neglecting the small velocity differences that appear between consecutive sections in the spinal canal. The associated errors can be expected to scale with the ratio of the intervertebral distance to the canal length.

In order to satisfy the flow rate shown in Fig. 2b, the cyclic pressure difference $p_{\text{C3}}-p_{\text{C4}}$ was adjusted by means of a proportional-integral-derivative controller.^1^ The adjustment process typically took between 50 and 60 flow cycles. To improve computational efficiency, a symmetry condition was applied on the plane dividing the right and left sections of the SSAS, thereby limiting calculations to one half of the domain.

The numerical integrations employed the commercial finite-volume solver ANSYS Fluent (2023 R1; Ansys Inc., Pennsylvania), with the SIMPLE (Semi-Implicit Method for Pressure-Linked Equations) algorithm used for velocity-pressure coupling with second-order accuracy in both space and time. We defined a time-implicit formulation with a time step of $0.01 \, \text{s}$, thereby dividing the time cycle, with period $T=1$ s, into 100 time steps. The iteration count per time step was fixed at 50 to ensure the residuals remained below $10^{-5}$. The solution was initialized at the beginning of the calculation by setting zero velocity throughout the fluid domain. The accompanying initial pressure head *p* must be uniform, as follows from the second equation in (4). Since the pressure enters only through its gradient, its initial value can be chosen arbitrarily (e.g. $p=0$). The solution was seen to evolve following the application of the C3-C4 pressure difference. A permanent periodic solution with ${\boldsymbol{v}}({\boldsymbol{x}},t)={\boldsymbol{v}}({\boldsymbol{x}},t+T)$ was established after about 65 cardiac cycles for the anatomically realistic geometry and after about 50 cycles for the unobstructed canal.

#### Solute transport

Once the permanent solution of the oscillatory velocity field in the cervical segment between the C3 and C4 vertebral levels was known, we addressed the problem of drug transport. This involved creating a fluid domain containing five successive cervical segments (see Fig. 1b). The numerical mesh used in solving the oscillatory flow was replicated five times to conform to the larger fluid domain. As indicated in Fig. 1b, the rostral and caudal boundaries correspond to the planes $x=x_r=1.6$ cm and $x_c=9.6$ cm, respectively. Drug dispersion was calculated by integrating the transport equation

5 $$\begin{aligned} \frac{\partial c}{\partial t} + {\boldsymbol{v}} \cdot \nabla c = \kappa \nabla ^2 c \end{aligned}$$

for the drug concentration *c*. Note that, unlike the reduced Eq. (1), convective transport in (5) is driven by the periodic CSF velocity ${\boldsymbol{v}}({\boldsymbol{x}},t)$, instead of the time-independent mean Lagrangian velocity ${\boldsymbol{v}}_L({\boldsymbol{x}})=(u_L,v_L,w_L)$, so that the resulting concentration $c({\boldsymbol{x}},t)$ displays rapid cyclic fluctuations, additional to the slow dispersion occurring in a longer time scale, whose description requires consideration of a large number of cardiac cycles. In the simulations, the value $\kappa = 7 \times 10^{-10}\, \mathrm {m^2/s}$ was selected for the molecular diffusivity of the drug, corresponding to a Schmidt number $\nu /\kappa = 1000$. Values of the Schmidt number exceeding one thousand are typical for most drugs injected into the SSAS, including Methotrexate, Thiotepa, Cytosine arabinoside, and EDTA-Ca, among others [52].

The inlet boundary at $x=1.6 \, \text{cm}$ and the outlet boundary at $x=9.6 \, \text{cm}$ (see Fig. 7) were treated as the exterior of the fluid domain, implying that any mass of the drug exiting the fluid domain would be lost. Since the focus here is on the description of drug dispersion, drug uptake was entirely neglected in the simulations. Correspondingly, the condition ${\boldsymbol{n}} \cdot \nabla c=0$ was applied on all bounding solid surfaces, with ${\boldsymbol{n}}$ denoting the unit vector normal to the impermeable surface pointing towards the liquid. As explained by Lawrence et al. [10], absorption of the drug could be accounted for in the simulations by incorporating modified boundary conditions on the inner and outer solid boundary surfaces (e.g. assuming the absorption rate to be linearly proportional to the local value of the concentration).

Consistent with the computation of ${\boldsymbol{v}}$, a time-implicit formulation was implemented with second-order temporal accuracy, using a time step of $0.01 \, \text{s}$. The third-order QUICK (Quadratic Upstream Interpolation for Convective Kinematics) method was employed for spatial discretization to minimize numerical diffusion and reduce associated errors. The iteration count per time step was fixed at 10 to ensure that the residuals remained below $10^{-5}$. The simulations of drug dispersion considered a bolus injected in the middle section of the cervical segment. The injection phase, whose description would necessitate additional information regarding the catheter geometry and release rate, was not considered. Instead, the numerical integrations were initiated at $t=0$ with an initial drug concentration described by the Gaussian function $c(x) = \exp [-(x-5.6)^2 / 0.5]$, centered at $x=5.6$ cm, where *x* is evaluated in centimeters. This distribution, uniform in the cross-sectional plane, corresponds to a bolus of volume $\int _{x_r}^{x_c} c(x) \, A(x) \, \text{d}x\approx 1.5$ mL, where *A*(*x*) is the open cross-sectional area.

### Implementation of the reduced model

Although DNS allows us to address the drug transport problem, the associated computational cost hinders applicability in clinical scenarios. Reduced-order models like that rigorously derived in our earlier work, Lawrence et al. [10], are more attractive for predictive purposes, in that the resulting transport equation, presented above in (1), involves computational times that are orders of magnitude smaller than those associated with DNS. The accuracy of the reduced model has been tested previously in connection with simplified spinal-canal models, Gutiérrez-Montes et al. [40], in which the characteristic streamwise length is much larger than the stroke length, that being one of the central assumptions underlying the derivation of (1). The present study aims at testing the applicability of the transport model to the description of drug dispersion in morphologically accurate geometries, using for that purpose the cervical canal in Fig. 1b. A natural question that arises is whether the mean Lagrangian velocity ${\boldsymbol{v}}_L$ continues to be the main driver for drug dispersion in the presence of nerve rootlets and denticulate ligaments, whose characteristic length is comparable to or smaller than the stroke length, so that the stroke-length to macroscopic-length ratio $\varepsilon$ is no longer a small quantity, thereby placing the system outside the range of conditions for which (1) is strictly applicable.

The reduced-order transport model (1) was implemented using a hybrid three-step approach. First, the time-periodic Eulerian velocity field ${\boldsymbol{v}}({\boldsymbol{x}},t)$ was calculated via DNS (see section Fluid flow). Second, the mean Lagrangian velocity ${\boldsymbol{v}}_L({\boldsymbol{x}})$ was evaluated by means of direct computation of fluid-particle trajectories advected by the Eulerian velocity field over one cycle. Third, the transport equation (1) was integrated to obtain the temporal evolution of drug transport, to be compared with the results of the DNS (section Solute transport). In this section we explain the technical details involved in steps two and three.

In the evaluation of the Lagrangian velocity ${\boldsymbol{v}}_L({\boldsymbol{x}})$, one should bear in mind that the expression (2), used in our previous works, is valid only when $\varepsilon \ll 1$. For the cervical canal analyzed here, that would require that the characteristic stroke volume $V_s$ of the cyclic motion be much smaller than the CSF volume $\int _{4.8}^{6.4} A(x) \, \text{d}x=V_{\mathrm{C3-C4}}=1.875$ mL contained in the vertebral section in the obstructed geometry shown in Fig. 2d, their ratio $\varepsilon =V_s/V_{\mathrm{C3-C4}}$ providing in this case the characteristic value of the parameter $\varepsilon$. Evaluation of the stroke volume with use of the flow rate given in Fig. 2b yields $V_s=\tfrac{1}{2} \int _t^{t+T} |Q| \text{d}t=0.823$ mL and $\varepsilon =V_s/V_{\mathrm{C3-C4}}=0.439$. Since the resulting value of $\varepsilon$ is not small, the accuracy of (2) is questionable, so that for increased accuracy the evaluation of ${\boldsymbol{v}}_L({\boldsymbol{x}})$ must rely on the direct computation of fluid-particle trajectories, as explained below.

For the periodic Eulerian velocity field ${\boldsymbol{v}}$, the trajectory ${\boldsymbol{x}}_p(t)$ of a fluid particle located at ${\boldsymbol{x}}_i$ at the initial time $t_i$ is determined by integration of

6 $$\begin{aligned} \frac{\text{d} {\boldsymbol{x}}_p}{\text{d} t}={\boldsymbol{v}}({\boldsymbol{x}}_p,t); \quad {\boldsymbol{x}}_p(t_i)={\boldsymbol{x}}_i. \end{aligned}$$

Since the velocity ${\boldsymbol{v}}$ is nonuniform, at the end of the cycle (i.e. at $t=t_i+T$) the particle does not return to its initial location but experiences instead a displacement given by

7 $$\begin{aligned} \delta {\boldsymbol{x}}({\boldsymbol{x}}_i,t_i)={\boldsymbol{x}}_p(t_i+T)-{\boldsymbol{x}}_i. \end{aligned}$$

As shown by Alaminos-Quesada et al. [53] in their analysis of particle drift with finite stroke lengths in the presence of obstacles, to evaluate the Lagrangian velocity ${\boldsymbol{v}}^*_L=(u^*_L,v^*_L,w^*_L)$ from the particle displacement, it is convenient to consider the average location of the fluid particle during the cycle

8 $$\begin{aligned} {\boldsymbol{x}}_o=\frac{1}{T} \int _{t_i}^{t_i+T} {\boldsymbol{x}}_p \text{d} t, \end{aligned}$$

and correspondingly define

9 $$\begin{aligned} {\boldsymbol{v}}^*_L({\boldsymbol{x}}_o)={\delta {\boldsymbol{x}}}/{T}. \end{aligned}$$

The above expression can be shown to reduce to ${\boldsymbol{v}}_L$, given in (2), for flows with stroke lengths much smaller than the characteristic macroscopic length. As shown for a compliant annular canal by Lawrence et al. [10], the procedure involves linearization of the trajectory equation (6) using the dimensionless stroke length $\varepsilon \ll 1$ as an asymptotically small parameter. Related insightful discussions can be found in the first paragraph of Subsection *Applications of the theory of steady streaming* (Section 5.13) in Batchelor [54].

To numerically evaluate ${\boldsymbol{v}}^*_L$ using the procedure delineated above we began by considering a large number of massless particles, emulating fluid elements, with initial locations ${\boldsymbol{x}}_i$ at the center of each cell of the entire computational mesh. We then followed the different fluid particles over an entire cycle and from their final positions evaluated their displacements $\delta {\boldsymbol{x}}$, which were then used in (9) to compute the value of ${\boldsymbol{v}}^*_L$. The resulting discrete distribution of velocity vectors ${\boldsymbol{v}}^*_L$ was interpolated linearly to the center of the different computational cells. Furthermore, to eliminate the dependence on the initial time $t_i$, the problem (6) was repeatedly integrated for different values of $t_i$ in the range $0< t_i < T$, with 20 different values of $t_i$ differing by 0.05*T* employed in the computations shown below. The resulting values were averaged to provide the final distribution of ${\boldsymbol{v}}^*_L({\boldsymbol{x}}_o)$.

Unlike the Lagrangian velocity evaluated from (2), which is solenoidal, i.e. $\nabla \cdot {\boldsymbol{v}}_L = 0$, when $\varepsilon$ is not infinitesimally small the Lagrangian velocity ${\boldsymbol{v}}^*_L$ evaluated from the trajectories is in general non-solenoidal as a result of the divergence or convergence of the fluid particles. This feature of the solution complicates its application to the computation of solute transport, as can be seen by writing the convective transport term in (1) in the conservative form ${\boldsymbol{v}}^*_L \cdot \nabla c=\nabla \cdot (c {\boldsymbol{v}}^*_L)-c \nabla \cdot {\boldsymbol{v}}^*_L$, where the second term, nonzero if $\nabla \cdot {\boldsymbol{v}}^*_L \ne 0$, represents a nonphysical distribution of sinks and sources that violates solute conservation.

The non-solenoidal nature of the Lagrangian-displacement field was addressed by Moffatt [55], who proposed its decomposition into solenoidal and non-solenoidal components (see Fig. 1 in Moffatt [55]). Following this author, we introduce the Helmholtz decomposition [56]

10 $$\begin{aligned} {\boldsymbol{v}}^*_L= {\boldsymbol{v}}_L + \nabla \Phi , \end{aligned}$$

where ${\boldsymbol{v}}_L$ is a solenoidal mean Lagrangian velocity field verifying $\nabla \cdot {\boldsymbol{v}}_L= 0$ and $\Phi$ is a potential scalar satisfying $\nabla \wedge (\nabla \Phi ) = 0$. Although such decomposition is known in the fluid mechanics community, as far as we know it has not been applied in the context of CSF flow. Applying the divergence operation to the expression (10) provides the Poisson’s equation

11 $$\begin{aligned} \nabla \cdot {\boldsymbol{v}}^*_L = \nabla ^2 \Phi , \end{aligned}$$

which can be integrated numerically to determine $\Phi$. The resulting function can be substituted into (10), finally yielding

12 $$\begin{aligned} {\boldsymbol{v}}_L = {\boldsymbol{v}}^*_L - \nabla \Phi , \end{aligned}$$

to be used in (1) when describing drug transport, thereby avoiding the anticipated solute-conservation issues associated with the nonsoleinoidal velocity ${\boldsymbol{v}}^*_L$.

The transport problem (1) was solved in ANSYS Fluent using the same mesh, boundary and initial conditions as those employed in the DNS of drug transport (section Solute transport). A time-implicit formulation was implemented with second-order temporal accuracy, using a time step of $1 \, \text{s}$, and a second-order upwind method for spatial discretization. The iteration count per time step was fixed at 10 to ensure that the residuals remained below $10^{-5}$.

## Discussion of illustrative results

The flow of CSF in the vertebral sections shown in Fig. 1c and d was characterized using DNS. The resulting velocity field was then used to compute mean Lagrangian velocities. Drug transport was investigated by quantifying the dispersion of a solute bolus released in the middle section of the cervical-canal model shown in Fig. 1b. The DNS results were used to evaluate the roles of molecular diffusion and anatomical obstacles in drug dispersion and also to assess the accuracy of the model equation (1) in relation to previously proposed models involving convective transport driven by the time-averaged Eulerian velocity [36, 37, 57].

### Oscillatory velocity field

The cross-sectional distribution of the axial velocity component *u* obtained for the canal with nerve rootlets and denticulate ligaments of Fig. 1c at $x=5.8 \, \text{cm}$ by integrating (4) is compared in Fig. 2d with the phase-contrast MRI velocity measurements. Results, presented at the moments of peak caudal and rostral flow rate, show excellent agreement. The largest velocities are found near the nerve rootlets, with peak values $u=-2.80 \, \mathrm {cm/s}$ (DNS) and $u=-2.97 \, \mathrm {cm/s}$ (MRI) for the rostral flow at $t=0$ and $u=5.04 \, \mathrm {cm/s}$ (DNS) and $u=5.02 \, \mathrm {cm/s}$ (MRI) for the caudal flow at $t=0.4 T$, respectively.

The pressure difference $p_{\text{C3}} - p_{\text{C4}}$ between the C3 and C4 levels (the upper and lower open boundaries of the fluid domain) corresponding to the imposed flow rate is shown in Fig. 2c. To quantify the influence of the nerve rootlets and denticulate ligaments on the pressure gradient, the results are compared with those obtained in computations using the obstacle-free canal. As can be seen, the pressure waveform is the same in both cases, but its amplitude is about 20% higher in the presence of anatomical obstacles, in agreement with previous observations [35]. Because of the nonlinear character of the flow, although the imposed flow rate has a zero mean value $\langle Q \rangle =0$, the associated pressure difference exhibits a small nonzero mean value given by $\langle p_{\text{C3}} - p_{\text{C4}} \rangle =0.001427 \, \text{Pa}$ (patent canal) and $\langle p_\text{C3} - p_{\text{C4}} \rangle =0.01387 \, \text{Pa}$ (obstructed canal).

### Secondary flow for small stroke lengths

**Fig. 3:**
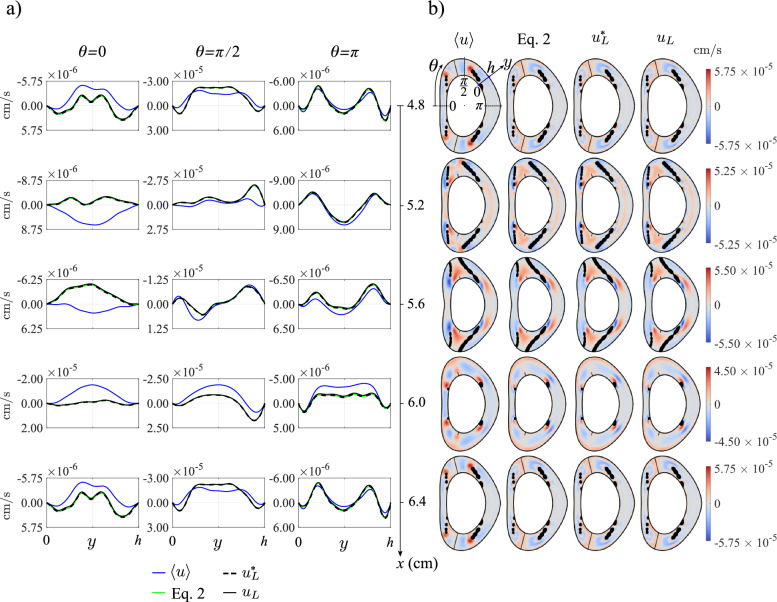
Comparison of $\langle u\rangle$, the axial component of the Lagrangian velocity given by Eq. (2), $u_L^*$ and $u_L$ in the long time scale for the obstructed geometry (corresponding to Fig. 1c) for a small stroke length, $\varepsilon = 0.00335$. **a)** Axial velocity profiles along the coordinate *y* between the pia mater and dura mater at various axial and azimuthal positions. The time-averaged Eulerian velocity, $\langle u\rangle$, is displayed in blue, the combined time-averaged Eulerian velocity and theoretical Stokes drift given by Eq. (2) is shown in green, the Lagrangian velocity given by Eq. (9), $u^*_L$, by black dashed lines and the solenoidal Langrangian velocity given by Eq. (12), $u_L$, by solid lines. **b)** Axial velocity contours of $\langle u\rangle$, the Lagrangian velocity given by Eq. (2), $u^*_L$ and $u_L$ at five different sections

The derivation of the reduced transport equation (1) assumes that the stroke length of the cyclic motion is small compared with the macroscopic length scale of the flow. Under those conditions, the mean Lagrangian velocity ${\boldsymbol{v}}_L$ can be computed precisely in terms of the Eulerian velocity ${\boldsymbol{v}}$ by application of the explicit equation (2), which involves the sum of the streaming velocity $\langle {\boldsymbol{v}} \rangle$ and the Stokes drift $\left\langle \int ({\boldsymbol{v}}-\langle {\boldsymbol{v}} \rangle ) \text{d}t \cdot \nabla ({\boldsymbol{v}}-\langle {\boldsymbol{v}} \rangle ) \right\rangle$. The applicability of (2) to the flow in the cervical region can be anticipated to be unlikely, because the stroke length associated with the flow rate given in Fig. 2b is fairly large, as measured by the associated value of $\varepsilon=V_s/V_{\rm C3-C4}\simeq 0.439$. Nevertheless, it is of interest to test the accuracy of (2), both to illustrate the differences between Eulerian and Lagrangian motion and to validate the numerical description of the CSF motion. To that end, flow computations were performed using a reduced flow rate, obtained by multiplying the physiologically accurate flow rate of Fig. 2b by a small factor 0.00763, yielding a small dimensionless stroke volume $\varepsilon=V_s/V_{\rm C3-C4}\simeq 0.00335$. The resulting velocity ${\boldsymbol{v}}$ was used to evaluate the streaming and Stokes-drift velocities. In addition, mean Lagrangian velocities ${\boldsymbol{v}}^*_L$ and ${\boldsymbol{v}}_L$ were computed from integrations of fluid-particle trajectories, as described above in section Implementation of the reduced model.

**Fig. 4:**
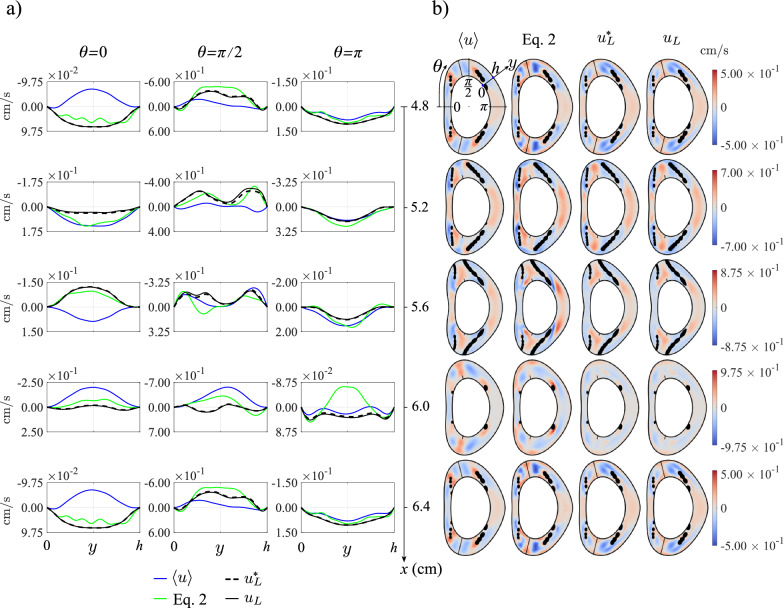
Comparison of $\langle u\rangle$, the axial component of the Lagrangian velocity given by Eq. (2), $u_L^*$ and $u_L$ in the long time scale for the obstructed geometry (Fig. 1c), corresponding to the physiologically accurate flow rate (Fig. 2b). **a)** Axial velocity profiles along the coordinate *y* between the pia mater and dura mater at various axial and azimuthal positions. The time-averaged Eulerian velocity, $\langle u\rangle$, is displayed in blue, the combined time-averaged Eulerian velocity and theoretical Stokes drift given by Eq. (2) is shown green, the Lagrangian velocity from Eq. (9), $u^*_L$, by black dashed lines and the solenoidal Langrangian velocity given by Eq. (12), $u_L$, by solid lines. **b)** Axial velocity contours of $\langle u\rangle$, the Lagrangian velocity given by equation (2), $u^*_L$ and $u_L$ at five different sections

Results corresponding to axial velocity distributions at five different sections along the intervertebral domain are shown in Fig. 3. Because of the flow periodicity, the results are identical at the entrance ($x=4.8$ cm) and exit ($x=6.4$ cm) sections. Besides color contours of cross-sectional distributions, represented in Fig. 3b, Fig. 3a shows transverse velocity profiles as a function of the distance to the pia mater, *y*, at three different azimuthal locations $\theta =0,\pi /2,\pi$, with $\theta$ measured from the anterior centerline, as indicated in the top left cross section of Fig. 3b. The comparison between the different velocity profiles indicate that the Lagrangian velocity computed with (2) is everywhere virtually indistinguishable from the two values $u^*_L$ and $u_L$ computed directly from the fluid-particle trajectories. By way of contrast, significant differences are found between the profiles of time-averaged Eulerian velocity $\langle u \rangle$, represented by the blue solid curves, and those corresponding to the mean Lagrangian velocity. The observed differences correspond to the Stokes drift, whose magnitude is comparable to that of the streaming velocity $\langle u \rangle$ in most places.

### The mean Lagrangian velocity

**Fig. 5:**
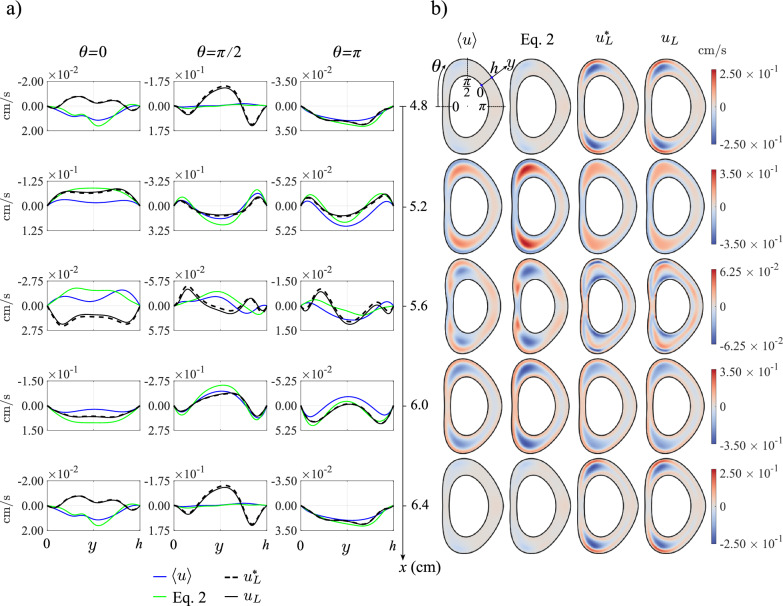
Same as in Fig. 4 for the patent (unobstructed) geometry

The secondary flow corresponding to the physiologically accurate flow rate of Fig. 2b is presented in Fig. 4 for the realistic (obstructed) geometry of Fig. 1c and in Fig. 5 for the patent canal of Fig. 1d. The plots show distributions of cycle-averaged axial velocity $\langle u \rangle$ and mean Lagrangian velocities, the latter including values computed with the expression (2) as well as those computed directly from the fluid-particle trajectories both before ($u_L^*$) and after ($u_L$) removing the nonsolenoidal component.

Observation of the figures reveals that the nonsolenoidal component of the mean Lagrangian drift $u_L^*-u_L$ is very small in all cases, with the largest differences in transverse profiles of $u_L^*$ and $u_L$ appearing in the anterior section $\theta =0$ in the presence of anatomical obstacles. As expected, the linearization that leads to the simplified equation (2) fails for the large value of $\varepsilon$ characterizing the flow in the cervical region, so that the Lagrangian velocity evaluated from (2) exhibits significant deviations from that determined from the trajectories. The results also illustrate the large differences that exist between the mean Eulerian velocity $\langle u \rangle$ and the mean Lagrangian velocity, in agreement with recent findings [38]. These relative differences, stemming from the spatial nonuniformity of the flow, are somewhat less noticeable in the posterior section $\theta =\pi$, where the velocity magnitude is very small.

The comparisons between the secondary flow corresponding to the anatomically realistic geometry (Fig. 4) and that of the patent geometry (Fig. 5) demonstrate the important effect of microanatomical features on the magnitude and spatial distribution of the secondary flow. In particular, axial speeds in Fig. 4 are seen to be significantly larger than those in Fig. 5 (note that the latter figure shows results over much smaller velocity ranges). The results are in agreement with previous computations pertaining to streaming velocities [24]. The amplification of the velocity magnitude is especially pronounced in the vicinity of nerve rootlets.

### Streamwise fluid-particle drift

**Fig. 6:**
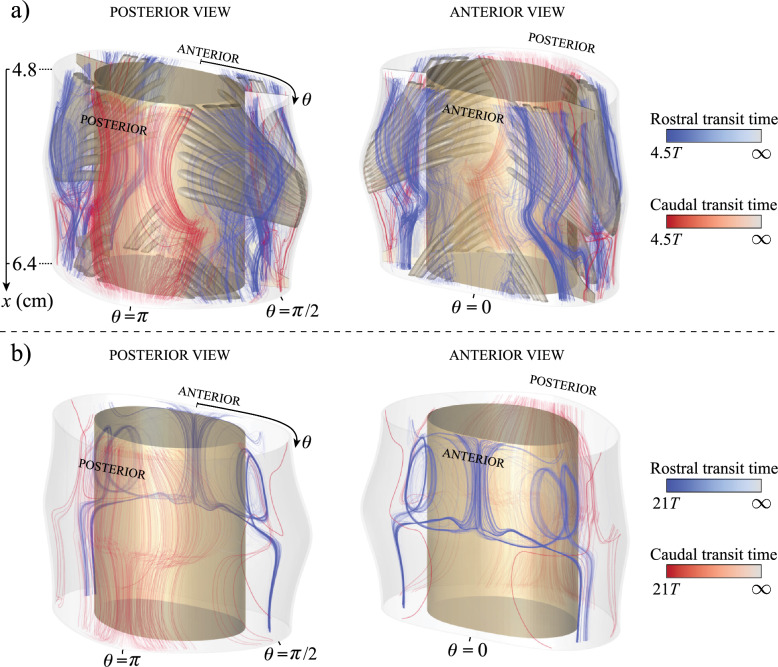
Fluid-particle trajectories from the solenoidal mean Lagrangian velocity, ${\boldsymbol{v}}_L$, in the long time scale within the cervical region of the spinal canal, specifically within the C3 and C4 vertebral levels for: **a)** the anatomically realistic geometry corresponding to Fig. 1c, and **b)** the patent geometry corresponding to Fig. 1d. Particles crossing caudally are shown in red and those crossing rostrally are shown in blue with brighter colors denoting shorter transit times, as indicated in the color bars

The important effect of microanatomical features on streamwise transport can be further evaluated by considering the fluid-particle trajectories associated with the solenoidal mean Lagrangian velocity ${\boldsymbol{v}}_L$. The computation was implemented numerically in ANSYS by tracking the motion of 5000 massless particles that were randomly distributed across the C3 and C4 cross sections at the initial time. Figure 6 shows only the trajectories of the particles that successfully traversed longitudinally the vertebral section during the time interval $0<t<4.5T$ for the obstructed canal and $0<t<21T$ for the patent canal, with particles crossing caudally shown in red and those crossing rostrally shown in blue, brighter colors denoting shorter transit times, as indicated in the color bars.

Comparative analysis of the results for patent and obstructed canals reveals that the latter geometry generates approximately four times more intervertebral connecting trajectories than the former. In the absence of obstacles, the Lagrangian drift exhibits recirculating patterns as in Gutiérrez-Montes et al. [40], reminiscent of those found in wavy-walled channels [58], thereby limiting the ability of fluid particles to traverse the fluid domain within the time interval considered. Conversely, the presence of nerve rootlets disrupts the recirculating motion, facilitating the formation of streamwise pathways that enhance particle dispersion. Notably, a pronounced caudal drift is observed along the posterior side (i.e., around $\theta =\pi$), while rostral drift is found predominantly on the lateral sides. These spatial flow patterns will be shown below to have an impact on the dispersion of the drug.

### Drug dispersion

**Fig. 7:**
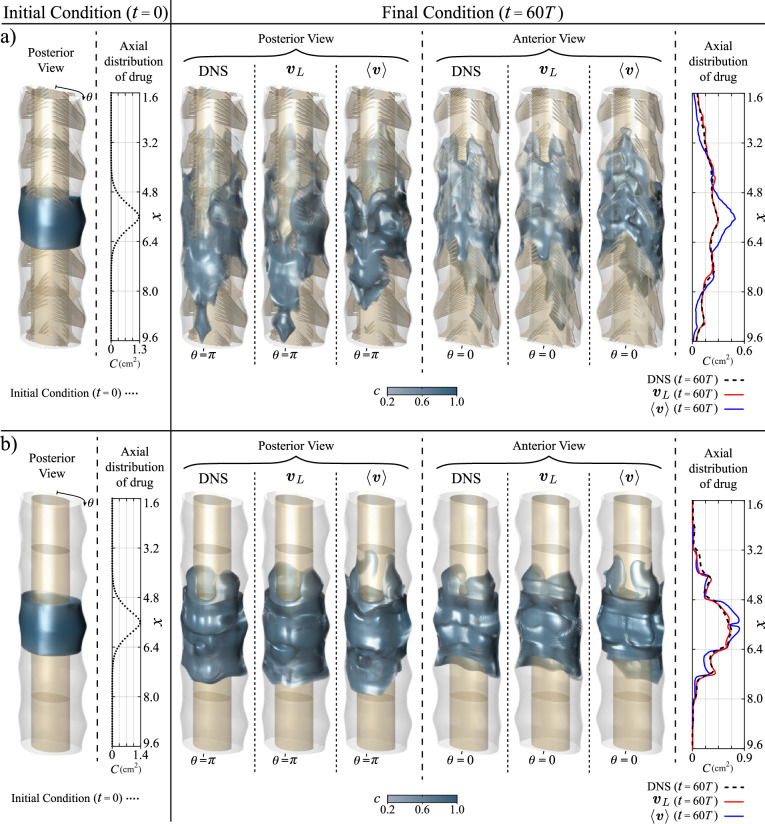
Drug distribution at $t= 60 T$ of an initial bolus described by the Gaussian function $c(x) = \exp [-(x-5.6)^2 / 0.5]$, uniformly distributed in the cross-sectional plane centered at $x=5.6$ cm (left panel). Posterior and anterior views of three-dimensional isosurfaces of drug concentration (central panels) are accompanied by side panels showing the longitudinal distribution of drug per unit canal length $C(x,t)=\int c \, \text{d}s$, obtained by integrating *c* across the canal cross section (right panel). Results integrating the model equation (1) with the Lagrangian velocity, ${\boldsymbol{v}}_L$, and the mean Eulerian velocity, $\langle {\boldsymbol{v}}\rangle$, are compared with those obtained by DNS for both, the patent geometry in **a)** and the anatomically realistic (unobstructed) geometry in **b)**

Results of integrations of the model equation (1) supplemented with the mean Lagrangian velocity of Figs. 4 and 5 are compared in Fig. 7 with those obtained by direct integration of the original transport equation (5) using the velocity obtained in the DNS flow computations described in section Oscillatory velocity field. Results obtained by integrating the reduced transport equation for the solute concentration using the mean Eulerian velocity, $\langle {\boldsymbol{v}}\rangle$, are also displayed for completeness. The figure shows the initial bolus as well as the drug distribution at $t=60T$ for the patent (Fig. 7b) and obstructed canals (Fig. 7a). The three-dimensional isosurfaces of drug concentration are accompanied by side panels showing the longitudinal distribution of drug per unit canal length $C(x,t)=\int c \, \text{d}s$, obtained by integrating *c* across the canal cross section.

The concentration distributions shown in Fig. 7a for the obstructed canal clearly demonstrate the predictive capability of the reduced model. The three-dimensional structure obtained via DNS, revealed by the isocontours of *c*, is closely described by the model equation (1), which adequately reproduces all relevant features, including the prominent caudal motion found on the posterior side, which is consistent with the Lagrangian drift patterns displayed in Fig. 6. Correspondingly, the departures of the predicted profiles of longitudinal drug distribution after 60 cycles *C*(*x*, 60*T*) from those determined via DNS are relatively small, as shown in the side panel of Fig. 7a. A quantitative measure of the associated error can be obtained by comparing the standard deviation $\sigma$ of the two distributions [59], which can be calculated using

13 $$\begin{aligned} \sigma ^2(t)=\frac{\int _{x_r}^{x_c} (x-{\bar{x}})^2 C(x,t) \, \text{d}x}{\int _{x_r}^{x_c} C(x,t) \, \text{d}x} \end{aligned}$$

with ${\bar{x}}(t)=\int _{x_r}^{x_c} x \, C(x,t) \, \text{d}x/\int _{x_r}^{x_c} C(x,t) \, \text{d}x$, the center of mass of the bolus, which is equal to 5.59 cm for the DNS and 5.47 cm for the model. These values indicate that, although the tracer dispersion occurs in the rostral and caudal directions, the center of mass of the drug remains close to the injection site, in agreement with the results reported in in-vivo experiments in pigs [60] and monkeys [61]. The computation of the standard deviations yields $\sigma =2.230$ cm for the DNS results and $\sigma =2.185$ cm for the model predictions. The resulting relative error of 2% is found to be very satisfactory, especially considering that the computational time associated with the integrations of the reduced equation (1) is about 125 times smaller than that involved in the integrations of (5).

As a way to test the accuracy of the previously proposed approximate models [36, 37], Fig. 7 also shows results obtained when the convective transport rate of the reduced transport equation (1) is evaluated with the mean Eulerian velocity $\langle {\boldsymbol{v}} \rangle$ replacing ${\boldsymbol{v}}_L$. As can be seen in Fig. 7a, the resulting drug concentration shows appreciable deviations from the DNS results, as the mean Eulerian velocity significantly underpredicts drug dispersion and also fails to capture many local features of the three-dimensional distribution. Correspondingly, the associated longitudinal distribution of concentration per unit canal length *C*(*x*) has a standard deviation $\sigma =1.577$ cm, significantly smaller than the value $\sigma =2.230$ cm computed via DNS, their relative difference being 28%.

Results corresponding to the unobstructed canal are given in Fig. 7b. After 60 cycles, the DNS reveal a compact drug distribution centered around the injection location. The associated standard deviation of the axial distributions of *C* is $\sigma =1.123$ cm, to be compared with the value $\sigma =2.230$ cm corresponding to the results of the obstructed canal. The limited drug dispersion observed in Fig. 7b is consistent with the weak longitudinal drift displayed in the plots of Fig. 6b, involving limited connecting trajectories and recirculating patterns. Clearly, the augmented secondary flow associated with the microanatomical features, visible in the trajectories shown in Fig. 6a, has a profound effect on drug transport. The enhanced convective transport aligns with the observations of Stockman [28], who reported a significant increase in longitudinal dispersion due to microanatomical features. Our findings emphasize the necessity of accurately modeling the canal morphology for increased accuracy of ITDD predictions.

For completeness, Fig. 7b includes predictions based on the reduced transport equation (1) and the alternative version employing $\langle {\boldsymbol{v}} \rangle$ instead of ${\boldsymbol{v}}_L$, alongside DNS results. The trends are consistent with those in Fig. 7a. Specifically, predictions using ${\boldsymbol{v}}_L$ closely match the DNS results, with only minor discrepancies.

### The effective hydrodynamic diffusivity

Following the classical theory of turbulent mixing by Taylor [62], one may use the DNS results of drug dispersion to determine the effective hydrodynamic diffusivity associated with the fluid motion in the cervical region according to

14 $$\begin{aligned} \kappa _H (t)=\frac{1}{2} \frac{{d} \sigma ^2}{{d} t}. \end{aligned}$$

For the specific transport problem considered here, equation (14) was evaluated using $\kappa _H (t)= [\sigma ^2(t)-\sigma ^2(0)]/(2 t)$. For the obstructed canal, $\kappa _H(t)$ was seen to increase with time to approach a nearly constant value, with minimum variations between $t=50T$ ($\kappa _H=3.944 \times 10^{-2}$ $\hbox {cm}^2$/s) and $t=60T$ ($\kappa _H=3.951 \times 10^{-2}$ $\hbox {cm}^2$/s). Similarly, for the unobstructed geometry we found $\kappa _H=8.633 \times 10^{-3}$ $\hbox {cm}^2$/s for $t=50T$ and $\kappa _H=8.577 \times 10^{-3}$ $\hbox {cm}^2$/s for $t=60T$. In this context, it is worth mentioning the work of Ayansiji et al. [59], who carried out in-vitro experiments to characterize $\kappa _H$ for different values of the CSF stroke volume and the oscillating frequency. For a cardiac period of $T=$ 1 s, their measurements yielded $\kappa _H=3.323 \times 10^{-2}$ $\hbox {cm}^2$/s for a stroke volume of $V_s=$ 0.5 mL and $\kappa _H=5.858 \times 10^{-2}$ $\hbox {cm}^2$/s for $V_s=$ 1 mL. In comparing with our results, corresponding to a stroke volume $V_s=0.823$ mL, one may use linear interpolation between these two experimental values to give $\kappa _H=4.960 \times 10^{-2}$ $\hbox {cm}^2$/s, which is in reasonably close agreement with the value $\kappa _H=3.951 \times 10^{-2}$ $\hbox {cm}^2$/s obtained numerically in our computations of the obstructed geometry.

Reduced models based on a simple diffusion equation incorporating an appropriately adjusted diffusivity have been proposed in recent work by Linninger et al. [63] as a basis to develop a distributed mechanistic pharmacokinetic model for analysis of ITDD procedures. Such models have the potential to become valuable computational tools in clinical practice, enabling efficient predictions of spatiotemporal drug dispersion along the neuraxis over time scales ranging from hours to weeks.

**Fig. 8:**
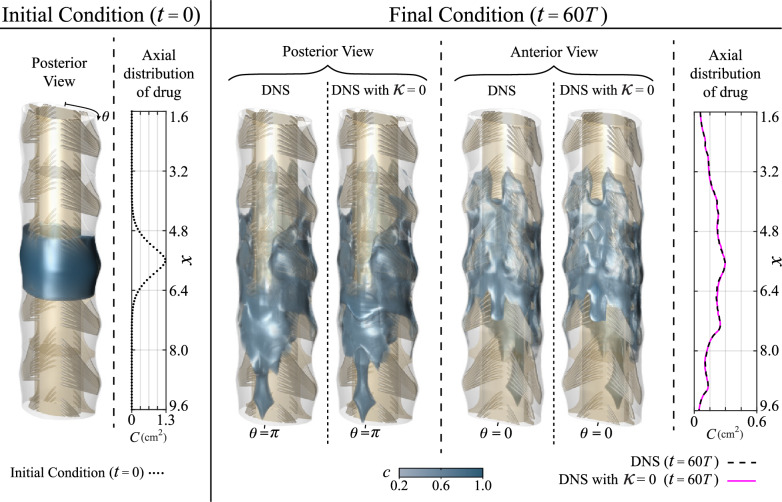
Comparison of drug distribution at $t= 60 T$ of an initial bolus described by the Gaussian function $c(x) = \exp [-(x-5.6)^2 / 0.5]$, uniformly distributed in the cross-sectional plane centered at $x=5.6$ cm (left panel), obtained numerically by integrating the species transport equation (5) with and without molecular diffusion. Posterior and anterior views of three-dimensional isosurfaces of drug concentration (central panels) are accompanied by side panels showing longitudinal distribution of drug per unit canal length $C(x,t)=\int c \, \text{d}s$, obtained by integrating *c* across the canal cross section (right panel)

### Effect of molecular diffusion

Given the extremely large value of the Schmidt number $\nu /\kappa \gtrsim 1000$ characterizing intrathecal drugs, the role of molecular diffusion is an open question. Its effect was found to be negligible in the lattice Boltzmann computations of Stockman [28] and was entirely neglected in the recent simulations of Khani et al. [37]. One may anticipate that streamwise diffusion has a negligible effect simply by comparing the drug molecular diffusivity $\kappa = 7 \times 10^{-6}\, \mathrm {cm^2/s}$ with the much larger value of the effective longitudinal hydrodynamic diffusivity evaluated with the DNS results $\kappa _H=3.951 \times 10^{-2}$ $\hbox {cm}^2$/s. Since the canal is relatively slender, the effect of molecular diffusion in the transverse direction (i.e. across the canal cross section) can be expected to be more pronounced. For small values of the dimensionless stroke length $\varepsilon$, previous order-of-magnitude arguments by Lawrence et al. [10] revealed that the transverse diffusive rate may affect the distribution of the drug provided that the value of $\varepsilon ^2 (\nu /\kappa )$ is not large. According to that reasoning, in the cervical region analyzed here, where $\varepsilon =0.439$ and $\varepsilon^2 (\nu/\kappa) \simeq 192.72 \gg 1$, molecular diffusion is expected to contribute negligibly also in the transverse direction.

To assess the relative importance of diffusive transport, the dispersion of the bolus was described numerically by integrating the species transport equation (5) with $\kappa =0$, thereby effectively removing molecular diffusion. The results of the diffusionless equation are compared in Fig. 8 with those of the full transport equation. As can be seen, both sets of results are virtually indistinguishable. As a measure of the error incurred by the diffusionless model, the local difference between the values of *C* of the two computations was integrated over the whole domain and the result was divided by the total amount of solute to give a relative deviation of 1.402%. Therefore, in accordance with the previous lattice Boltzmann computations by Stockman [28], our DNS results support the notion that molecular diffusion is rather inconsequential for drug dispersion, with convective transport driven by the mean Lagrangian drift emerging as the primary transport mechanism.

### Limitations

The current analysis omitted important effects that may influence drug dispersion in the SSAS. For instance, nerve rootlets and denticulate ligaments were included in our anatomical model of the SSAS, but trabeculae were not considered, because of their sparse distribution in the cervical region [25]. As suggested in previous studies [34], these additional microanatomical structures might affect flow and transport in other parts of the spinal canal, so that incorporating them into future SSAS models could enhance predictive accuracy.

While our work considered only cardiac-driven flow oscillations, which are prevalent in the cervical region, the model could be extended to incorporate velocity fluctuations induced by breathing, as done in recent numerical computations [37]. Since these fluctuations are known to dominate the CSF dynamics in the lumbar region [7], their inclusion appears to be critical in future predictive efforts targeting drug dispersion from typical L3-L4 injection sites.

As demonstrated in Appendix A, the displacement of the dura has a negligibly small effect on the local mean Lagrangian flow, allowing drug dispersion along a short stretch of the canal to be studied using a rigid model, as done in the present work. Nevertheless, since the spinal canal is closed at its sacral end, compliance is crucial for enabling fluid motion. Therefore, future modeling efforts considering the entire canal must account for the deformation of the dura membrane. The spatial nonuniformity associated with the streamwise variation of the resulting velocity field, including cardiac-driven stroke volumes that decay caudally [23], can be expected to promote secondary flow, thereby further increasing drug dispersion rates. Future modeling efforts addressing the entire neuraxis should also incorporate pharmacokinetics phenomena such as drug enzymatic decay, tissue uptake and clearance by the blood [59, 63] along with buoyancy-driven flow stemming from density differences between the CSF and the drug [41, 42]. These developments should be ultimately validated by comparison with measurements from in-vivo and in-vitro experiments.

The reduced transport model presented above accurately describes the slow evolution of a drug bolus but cannot be used to investigate the fast injection phase leading to the formation of the initial drug distribution. This limitation arises because the injection velocities are typically larger than those of the mean Lagrangian drift and also because the associated injection time scales, on the order of seconds, are much shorter than those governing drug dispersion. Consequently, the effects of injection protocols and parameters—known to be significant [37, 64]—should be studied using alternative methods, such as direct numerical simulations and in-vitro experiments.

## Concluding remarks and future prospects

This paper used numerical simulations to investigate the dispersion of a neutrally buoyant (isobaric) drug in the spinal canal. One of the objectives was to test the predictive capability of the reduced transport equation (1), involving convective transport driven by the mean Lagrangian drift. The equation was originally derived by Lawrence et al. [10] using a rigorous asymptotic description in which the stroke length of the CSF oscillatory flow is assumed to be smaller than the characteristic streamwise length of the canal geometry. However, because actual CSF flow often exhibits stroke lengths comparable to the intervertebral distance, the applicability of the reduced-order model under realistic spinal-flow conditions remains uncertain, motivating this work. Model validation was conducted by analyzing drug transport in the cervical spine using high-fidelity, MRI-informed DNS and comparing the results with those from the reduced-order model. Additionally, the influence of microanatomy was examined by evaluating the impact of removing nerve rootlets and denticulate ligaments from the realistic anatomical model.

Specific attention was given to the numerical evaluation of the mean Lagrangian velocity. The analysis revealed that the asymptotic expression (2) is only accurate for small stroke lengths, as it should, so that the evaluation of ${\boldsymbol{v}}_L({\boldsymbol{x}})$ must in general rely on direct computations of fluid-particle trajectories (see Figs. 3, 4, 5). The analysis of the role of the microanatomy (Fig. 6) reveals that nerve rootlets and denticulate ligaments strongly influence CSF velocities, increasing the associated drug dispersion rate by a factor exceeding four, in agreement with previous works [24, 27, 28, 34, 37]. The spatio-temporal drug distribution obtained by integrating (1) was found to compare very well with that obtain via DNS (Fig. 7), demonstrating that the reduced model is able to accurately describe drug dispersion at a fraction of the computational cost required by the DNS. In particular, the computational time associated with the integrations of the reduced equation (1) is over two orders of magnitude smaller than that involved in the integrations of (5). The alternative transport equation obtained by replacing the mean Lagrangian velocity ${\boldsymbol{v}}_L({\boldsymbol{x}})$ with the cycle-averaged Eulerian velocity $\langle {\boldsymbol{v}} \rangle$, an approximation used in previous studies [36, 37], was found to severely underpredict drug dispersion, thereby highlighting the important differences between both velocity fields, pointed out in recent work [38]. The role of molecular diffusion was also tested (Fig. 8), and our results indicate that setting it to zero has a negligible influence on drug dispersion. Consequently, the diffusionless version of the reduced transport equation (1), ${\partial c}/{\partial t} + {\boldsymbol{v}}_L \cdot \nabla c =0$, appears to be an attractive alternative for ITDD quantification. Numerical methods specifically tailored to describe Lagrangian particle dispersion can be instrumental in enabling future computations [65]. Finally, we used the DNS results to evaluate the effective hydrodynamic diffusivity (14). The results can be incorporated into one-dimensional diffusion equations describing axial drug dispersion, a simple modeling strategy proposed in previous works [59, 63]. The computational framework provided by the present study can be readily extended to incorporate additional effects, including drug baricity and pharmacokinetics. These future modeling efforts could clearly benefit from accompanying in-vitro experiments considering realistic representations of the SSAS anatomy, as those performed recently [59].

## Figures and Tables

**Fig. 9:**
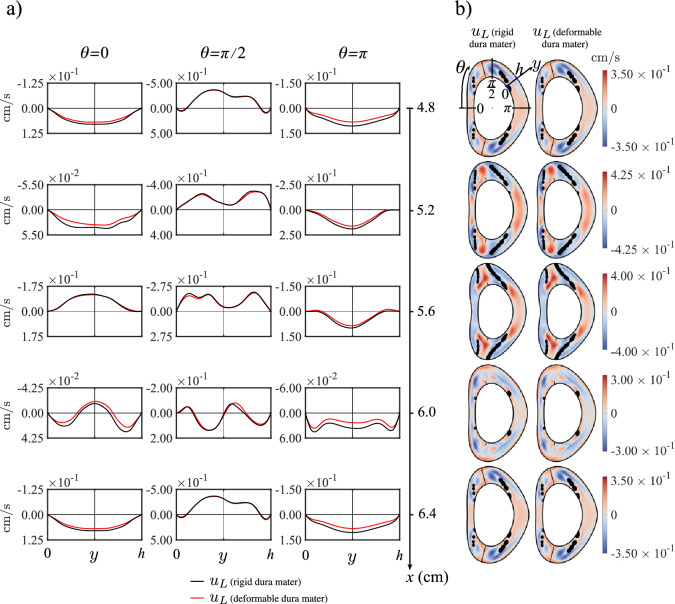
Comparison of the axial component of the mean Lagrangian velocity, $u_L$, for the obstructed geometry (Fig. 1c), corresponding to the physiologically accurate flow rate (Fig. 2b), obtained from numerical simulations considering a rigid and a deformable model. **a)** Axial velocity profiles along the coordinate *y* between the pia mater and dura mater at various axial and azimuthal positions. **b)** Axial velocity contours of $u_L$ at five different sections

## Data Availability

The raw data are available from the corresponding author on reasonable request.

## Appendix A: Influence of dura mater movement on the mean Lagrangian velocity field

In each cardiac cycle, the induced intracranial pressure fluctuations drive a small volume of CSF periodically in and out of the compliant SSAS, generating an oscillatory motion of CSF along the spinal canal. This volume is accommodated by the compression of the venous and fatty tissue in the epidural space that surrounds the dural sac [4]. The stroke volume is known to decrease monotonically in the caudal direction, to eventually be null at the sacral end of the spine. In our computations, the dura membrane has been considered rigid, assuming that the local effect of the stroke volume variation would be negligible in our model section. In the present appendix, we evaluate the effect of modeling the dura membrane deformation on the velocity field. To that aim, Fig. 9 shows a comparison of the solenoidal axial mean Lagrangian velocity, $u_L$, obtained for the cervical canal in Fig. 1b considering the dura membrane as a rigid wall and as a deforming wall. In the deformable case, the simulations have been carried out implementing a dynamic mesh that accounts for the motion of the outer boundary of the model, compatible with a monotonically decreasing stroke volume in the SSAS displayed in Fig. 1a, of total length 59 cm. The flow rate imposed at the inlet, was adjusted to guarantee that the flow rate in Fig. 2b was obtained at the midpoint between the C3 and C4 vertebral levels, i.e. at $x=5.6$ cm. At the outlet, a reference pressure was prescribed. As observed in Fig. 9, the profiles and contours of $u_L$ obtained considering the dura as a rigid and a deforming wall almost coincide, demonstrating that the local effect of the dura motion can be neglected in the present case.

## Abbreviations

$$\rho$$: Density (kg/m$$^3$$)
$$\nu$$: Kinematic viscosity (m$$^2$$/s)
$p$: Pressure head (Pa)
${\boldsymbol{v}}$: Velocity vector (cm/s)
$\langle {\boldsymbol{v}} \rangle$: Time-averaged Eulerian velocity (cm/s)
${\boldsymbol{v}}_L$: Solenoidal mean Lagrangian velocity (cm/s)
${\boldsymbol{v}}^{*}_{L}$: Mean Lagrangian velocity (cm/s)
$$t$$: Time (s)
$$T$$: Period (s)
$\omega$: Angular frequency (rad/s)
$$\kappa$$: Molecular diffusivity (m$$^2$$/s)
$\kappa _H$: Effective hydrodynamic diffusivity (m$^2$/s)
$$c$$: Drug concentration
$C$: Drug concentration per unit canal length (cm$^2$)
$V_s$: Stroke volume (cm^3^)
$$\varepsilon$$: Small parameter
$$\Phi$$: Potential scalar (m^2^/s)
${\boldsymbol{x}}$: Position vector (cm)
CSF: Cerebrospinal fluid
SAS: Subarachnoid space
CSAS: Cerebral subarachnoid space
SSAS: Spinal subarachnoid space
MRI: Magnetic resonance imaging
DNS: Direct numerical simulation
ITDD: Intrathecal drug delivery
TE: Echo time (ms)
TR: Repetition time (ms)
FOV: Field of view (mm$^2$)
SIMPLE: Semi-implicit method for pressure-linked equations
QUICK: Quadratic upstream interpolation for convective kinematics

## Footnotes

1 A proportional-integral-derivative (PID) controller is a feedback control algorithm that iteratively calculates the error between a target setpoint and a measured output. Consequently, corrections are applied based on proportional, integral, and derivative procedures on a variable to minimize the error [51].
